# Chancen und Herausforderungen der zunehmenden Digitalisierung der Lehre im Fach Anästhesiologie aus Sicht der Studierenden

**DOI:** 10.1007/s00101-022-01102-1

**Published:** 2022-03-03

**Authors:** S. Hunck, K. Engelhard, P. Mildenberger, S. Kurz

**Affiliations:** 1 Klinik für Anästhesiologie, Universitätsmedizin, Johannes Gutenberg-Universität Mainz, Mainz, Deutschland; 2 Institut für medizinische Biometrie, Epidemiologie und Informatik, Universitätsmedizin, Johannes Gutenberg-Universität Mainz, Mainz, Deutschland; 3 Rudolf Frey Lernklinik, Universitätsmedizin, Johannes Gutenberg-Universität Mainz, Langenbeckstr. 1, 55131 Mainz, Deutschland

**Keywords:** Medizinische Lehre, Digitale Lehre, Anästhesiologische Lehre, Blended Learning, COVID-19, Medical education, e-Learning, Anaesthesiologic teaching, Blended Learning, COVID-19

## Abstract

**Hintergrund:**

Die COVID-19-Pandemie hat die medizinische Lehre weltweit verändert. Seit dem Sommersemester 2020 stehen digitale Lehrformate im Fokus, deren Einsatz zuvor in Intensität und Form in Deutschland sehr unterschiedlich ausgeprägt war.

**Ziel der Arbeit:**

Die vorliegende Untersuchung stellt die Chancen und Herausforderungen des Einsatzes verschiedener digitaler Lehrformate in der Anästhesiologie aus der Sicht der Studierenden dar.

**Methode:**

Medizinstudierende der Semester 5–9 im Fachgebiet Anästhesiologie wurden anhand eines 5‑Punkte-Likert-skalierten Fragebogens zu ihren Einstellungen und Erfahrungen mit der digitalen Lehre im Sommersemester 2020 befragt. Untersuchte Lehrformate waren digital aufgezeichnete Vorlesungen sowie Video-Online-Seminare und Praktika.

**Ergebnisse:**

An der vorliegenden Studie nahmen 141 Studierende teil. Insgesamt haben 80,9 % der Studierenden mehr aufgezeichnete Vorlesungen online angehört, als sie Präsenzvorlesungen besucht hätten, und 84,3 % würden dies auch zukünftig so machen. Obwohl die Studierenden digitale Vorlesungen und Video-Online-Seminare für die Wissensvermittlung als geeignet beurteilten, besteht für die Zukunft der Wunsch, diese zur Wissensvertiefung wieder durch mehr praktischen Präsenzunterricht in Kleingruppen zu ergänzen.

**Schlussfolgerung:**

Als Vorteil digitaler asynchroner Formate unterstreicht die Studie die Option des zeit- und ortsunabhängigen individuellen Lernens. Als klarer Nachteil dieser Formate ist die mangelnde Interaktion von Studierenden und Dozierenden zu nennen. So erscheint es sinnvoll, die im Rahmen der COVID-19-Pandemie forcierte Digitalisierung für die Wissensvermittlung der für die Anästhesiologie relevanten Inhalte weiterauszubauen und im Sinne einer Blended-Learning-Strategie zu optimieren.

## Abstract (en)

**Background:**

The coronavirus pandemic caused a sudden change in medical education worldwide and induced a shift towards digital teaching. Previously, most courses were organized for students in physical presence on campus, while a few institutions already complemented these with blended learning methods, combining digital teaching with clinical presence; however, the use of digital teaching was heterogeneous, ranging from the use of PowerPoint slides to the application of virtual patients and telemedicine.

**Objective:**

This study aimed to identify challenges and opportunities arising from the different tools used in digital teaching, such as recorded lectures or online seminars, and the role of hands-on clinical experience. In addition, the study examined student attitudes and experiences with the nearly all-digital semester beginning in spring 2020. These findings may help to better understand the impact of digital teaching on students and provide guidance on how to optimize digital medical teaching in the future.

**Methods:**

A questionnaire with a 5-point Likert scale was developed to assess students’ experiences with digital teaching at the onset of the COVID-19 pandemic in a cross-sectional study. The questionnaire was distributed to all medical students at the University of Mainz, Germany, who attended an anesthesiology course with asynchronously recorded lectures replacing traditional on-campus lectures during the pandemic. A total of 766 students in 4 courses met these criteria and received the online questionnaire for anonymous and voluntary response.

**Results:**

A total number of 141 students responded, resulting in a response rate of 18.4%. Almost all students had access to the appropriate technology to participate in digital courses and 80.9% indicated seeing more recorded lectures compared to previous face-to-face lectures on campus. Moreover, 67.4% reported that they paused the asynchronous lectures to look up additional information and 95.7% stated that they appreciated the possibility to view lectures regardless of time and location. Hence, 84.3% said they would prefer digital lectures over face-to-face lectures in the future; however, 28.4% missed the interaction with teachers during lectures. Thus, 94.3% of the students appreciated the online seminars in terms of theoretical knowledge but the majority missed the practical aspects in the courses. Overall, 78.0% responded that the digital availability of material and digital teaching formats helped them to enhance their independent learning abilities.

**Conclusion:**

This study highlights several opportunities and challenges associated with digital teaching. The availability of prerecorded lectures at any time and place seems to facilitate access and increases student motivation. It also promotes individualization of student learning. Main disadvantages are the lack of communication with teachers and other students as well as the missing opportunity to reinforce the theoretical knowledge through application in practical courses, which, however, are essential for medical education. Therefore, a possible solution in the future could be the introduction of the “inverted classroom” concept, as it focuses on application and deepening of clinical skills in interactive classes, while the theoretical knowledge acquisition is taught in a digital teaching environment. The present study proposes the introduction of blended learning concepts to enhance the benefits of digital teaching while minimizing the identified disadvantages.

Die COVID-19-Pandemie und die resultierende Notwendigkeit der sozialen Distanzierung haben die medizinische Lehre weltweit verändert. Die traditionelle Präsenzlehre musste ab dem Sommersemester 2020 weitestgehend digitalen Lehrformaten weichen, deren flächendeckender Einzug in die medizinische Lehre eine enorme Veränderung für Studierende und Dozierende weltweit darstellt. Die Chancen und Herausforderungen verschiedener digitaler Lehrformate für die Studierenden sollen am Beispiel der Universitätsmedizin Mainz im Fachbereich Anästhesiologie analysiert werden.

## Hintergrund

Der Ausbruch von SARS-CoV-2, welcher am 11.03.2020 von der WHO zur Pandemie erklärt wurde, hat die medizinische Lehre weltweit in sehr kurzer Zeit verändert und die meist vollständig digitale Lehre erzwungen [3, 20, 25]. In der Vergangenheit wurde der Einsatz digitaler Lehrmethoden in der Medizin in Deutschland sehr unterschiedlich gehandhabt. Die Bandbreite reichte von der Verwendung einer Power-Point-Präsentation (Microsoft Corporation, Albuquerque, NM, USA) über das Aufzeichnen von Vorlesungen bis hin zum Einsatz virtueller Patienten und der Beschäftigung mit der Telemedizin. Diese Formate wurden in erster Linie zur Unterstützung der Präsenzlehre eingesetzt [12]. In der Anästhesiologie gab es bereits im Jahr 2000 erste Projekte, die studentische Ausbildung im Rahmen der Notfallmedizin mit Simulationen digital zu unterstützen [9]. Eine flächendeckende Strategie zum Einsatz digitaler Formate in der medizinischen Lehre hat es in Deutschland nicht gegeben.

Bei der Betrachtung dieser Thematik sind klare Begriffsdefinitionen von Bedeutung. Die Präsenzlehre bezeichnet in dieser Arbeit alle Lehrformate, bei der die Dozierenden und Studierenden physisch anwesend sind. Die digitale Lehre beschreibt den synchronen oder asynchronen Einsatz von Lehrformaten, die ausschließlich online stattfinden. Dies kann beispielsweise zur Verfügung gestelltes Material zum Selbststudium sein, aber auch ein online stattfindendes Seminar. Das Konzept des Blended Learning kombiniert die digitale Lehre mit der Präsenzlehre [7, 13, 16].

Die kurzfristige Umstellung auf eine rein digitale Lehre im Frühjahr 2020 hat aufgrund der bisherigen Heterogenität der Lehrformate unterschiedlich gut funktioniert. Was zuerst vielerorts als „notfallbedingte Fernlehre“ [18] entstanden ist, sollte als Chance gesehen werden, die Herausforderungen und Möglichkeiten der digitalen Lehre für Studierende und Dozierende zu explorieren und für die zukünftige Gestaltung der medizinischen Lehre einzusetzen [4, 18, 24]. Zielsetzung dieser Studie ist es, mögliche Herausforderungen und Chancen der digitalen Lehre sowie Implikationen für die zukünftige Lehre im Fachgebiet Anästhesiologie aus Sicht der Studierenden aufzuzeigen.

## Material und Methoden

Zur Erhebung der Einstellungen und Erfahrungen der Studierenden der Universitätsmedizin Mainz mit der digitalen Lehre im „Corona-Sommersemester 2020“ wurde zur Durchführung einer Querschnittstudie ein Online-Fragebogen mit LimeSurvey (LimeSurvey GmbH, Hamburg, Deutschland) entwickelt. Der Fragebogen beinhaltete 20 Fragen in 6 Unterkategorien. Im Rahmen der explorativen Meinungsforschung bewerteten die Studierenden v. a. Aussagen anhand einer 5‑Punkte-Likert-Skala mit den Dimensionen „stimme nicht zu“ bis „stimme zu“. Die Kategorien „stimme eher zu“ und „stimme zu“ wurden in der Auswertung als Zustimmung gewertet. Die Unterkategorien erfassten neben allgemeinen Daten die Einstellungen und Erfahrungen der Studierenden zu den Themenbereichen Vorlesung, Seminar und Praktikum sowie der Zukunft der anästhesiologischen Lehre. Vorlesungen, Seminare und ein Teil des Praktikums wurden an der Universitätsmedizin Mainz ausschließlich digital angeboten. In der Bibliothek der Universitätsmedizin Mainz konnten Tablets ausgeliehen werden, um jedem Studierenden einen digitalen Zugang zu ermöglichen.

In Mainz wurde das Format der 45-minütigen anästhesiologischen Präsenzvorlesung in 20-minütige digitale Impulsvorträge umgewandelt, die sich auf die zuvor festgelegten, wesentlichen Lernziele fokussierten. Diese Impulsvorträge wurden in der Studie definiert als digital aufgezeichnete Vorlesungen (DAVL), die vom Dozierenden vorab aufgenommen und den Studierenden asynchron auf dem Lernmanagementsystem (LMS) Moodle^TM^ (Moodle Pty Ltd, West Perth, Australia) bereitgestellt wurden. Neben den Vortragsfolien stellten Dozierende weiterführende Informationen z. B. in Form von Verlinkungen zu AMBOSS-Artikeln (AMBOSS GmbH, Berlin, Deutschland), Büchern oder wissenschaftlichen Veröffentlichungen bereit. Bei Unklarheiten konnten Dozierende per E‑Mail kontaktiert werden. Am Ende jedes Impulsvortrags wurden Multiple-Choice-Fragen eingestellt, nach deren Beantwortung der Vortrag als „angesehen“ galt.

Einige Seminare wurden in digitaler Form als Video-Online-Seminare (VOS) abgehalten. Dieser Begriff beschreibt ein synchrones Seminar mit Interaktion zwischen Dozierenden und Studierenden über eine digitale Plattform. An der Universitätsmedizin Mainz wurden Microsoft Teams (Microsoft Corporation, Albuquerque, NM, USA) und Big Blue Button (open source web conferencing system) verwendet. VOS bieten neben der Interaktion über Audio und Video u. a. einen Chat für Fragen sowie Möglichkeiten für Abstimmungen oder Quizfragen.

Zusätzlich zu den bereits genannten DAVL und VOS wurden im Sinne einer Blended-Learning-Strategie eLearning-Module angeboten, in denen durch Lehrvideos, Texte und Aufgaben zur Gesprächsführung mit Patientinnen und Patienten und zu Abläufen im OP weitere Themenbereiche abgedeckt werden konnten. Unter strengen Hygieneauflagen konnten teilweise praktische Präsenztermine in Kleingruppen stattfinden.

Die Zielgruppe der Studierenden wurde so festgelegt, dass alle Kurse der Anästhesiologie eingeschlossen wurden, die mindestens das Lehrformat der Vorlesung enthielten.

Daraus ergaben sich die in Tab. 1 dargestellten Kurse pro Semester.

**Table 1:** 

*5. Semester*	Digitale asynchrone Vorlesung Anästhesiologie 1
*7. Semester*	Digitale asynchrone Vorlesung Anästhesiologie 2
	eLearning-Modul zur Praktikumsvorbereitung
	3‑tägiges OP-Praktikum Anästhesiologie 2
*8. Semester*	Digitale asynchrone Vorlesung Q14 Schmerzmedizin
	Digitale asynchrone Online-Seminare Q14 Schmerzmedizin
	eLearning-Module als Ersatz für Kleingruppenunterricht in Q14
*9. Semester*	Digitale asynchrone Vorlesung Q8 Notfallmedizin
	Digitale asynchrone Online-Seminare Q8 Notfallmedizin
	4‑stündiger Kleingruppenunterricht „Megacodetraining“ Q8 Notfallmedizin

*Q14* Querschnittsfach 14 Schmerzmedizin, *Q8* Querschnittsfach 8 Notfallmedizin

Nach Auswahl des belegten Semesters zu Beginn der Umfrage wurden den Studierenden nur die für den Kurs zutreffenden Fragenblöcke angezeigt. Der Fragebogen wurde per E‑Mail an die insgesamt 766 eingeschriebenen Studierenden der genannten Kurse mit einem erläuternden Einladungstext geschickt. Zusätzlich erfolgte die Vorstellung der Umfrage in den sozialen Gruppen der Studierenden, über die zusätzlich im 2‑wöchentlichen Abstand insgesamt 2 Erinnerungen zur Teilnahme an der Umfrage versendet wurden. Das anonyme Ausfüllen der Umfrage zum Ende des Sommersemesters 2020 im Zeitraum Juli und August 2020 erfolgte auf freiwilliger Basis.

Die deskriptive Auswertung der Fragebogen erfolgte mit SPSS Statistics (IBM, Armonk, NY, USA, Version 23).

## Ergebnisse

**Figure 1:**
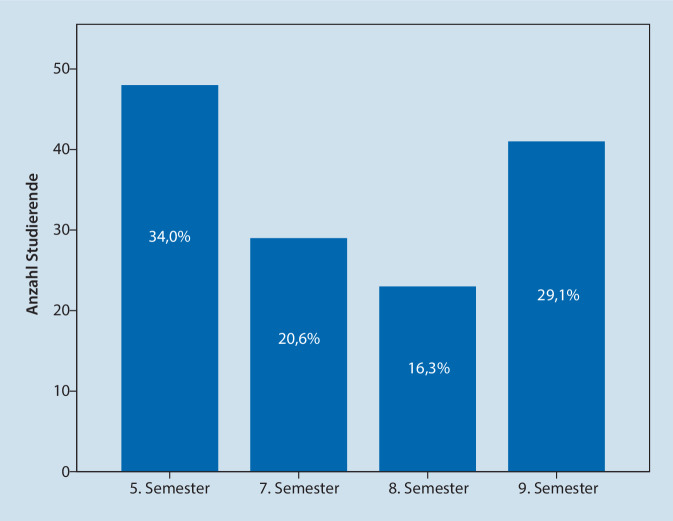

Nach Abschluss der Umfrage lagen 141 (18,4 %) vollständig ausgefüllte Fragebögen vor. Im 5. und 9. Semester war die Anzahl der Studierenden, die den Fragenbogen ausgefüllt haben, am höchsten (Abb. 1). Das Alter der Teilnehmenden reichte von 20 bis 35 Jahren mit einem Mittelwert von 26 Jahren. Insgesamt gaben 137 (97,9 %) Studierende an, eine adäquate technische Ausstattung für die digitale Lehre zu besitzen. In der folgenden Analyse werden die Studierenden als Gesamtheit betrachtet und die Ergebnisse nach Lehrformat aufgeteilt dargestellt.

### Digital aufgezeichnete Vorlesung

Von den teilnehmenden Studierenden gaben 122 (86,5 %) t an, mindestens die Hälfte der Vorlesungen ihrer Vorlesungsreihe bearbeitet zu haben, 76 (53,9 %) sahen alle Vorlesungen an. Darüber hinaus pausierten 95 (67,4 %) Studierende die Vorlesungsaufzeichnung, um sich weitere Informationen anzulesen und ihr Wissen zu vertiefen, 53 (37,6 %) Studierende riefen Vorlesungen mehrfach ab. Zur Selbsteinschätzung der Studierenden wurden Quizfragen in die Impulsvorträge eingebaut, die von 111 (79,9 %) als hilfreich beschrieben wurden, um das Erlernte zu festigen.

**Figure 2:**
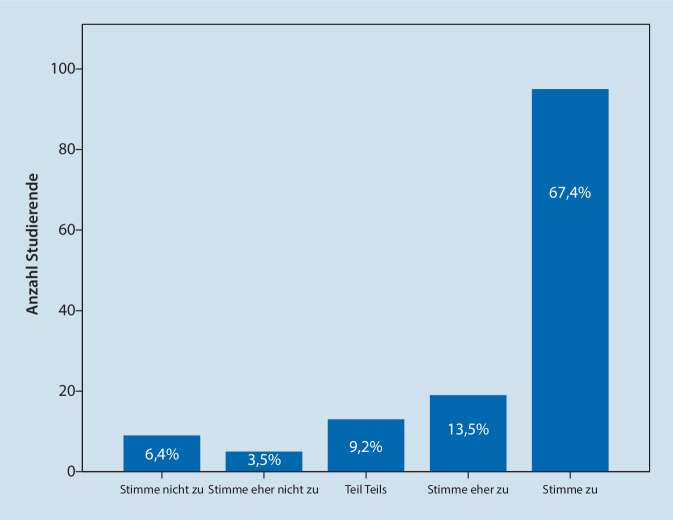

Insgesamt schätzten 134 (95,7 %) Studierende die Möglichkeit des orts- und zeitunabhängigen Lernens im eigenen Tempo und 114 (80,9 %) Studierende gaben an, mehr DAVL besucht zu haben, als es bei Präsenzvorlesungen der Fall gewesen wäre (Abb. 2). Letztendlich stimmten 101 (71,6 %) Studierende zu, dass sie die Lerninhalte digital umfassender und teilweise (66,6 %) schneller verstehen konnten als in einer Präsenzvorlesung.

Zusätzlich gaben 88 von 121 (72,7 %) Studierenden an, dass sie in fortgeschrittenen Semestern weiterhin DAVL aus bereits absolvierten Semestern zur Prüfungsvorbereitung konsultieren würden und 122 (87,1 %) Studierende beschrieben sich als motiviert für das Bearbeiten von DAVL.

Ein Anteil von 50 (35,5 %) Studierenden hätte zumindest teilweise gerne Rückfragen gestellt und 40 (28,4 %) Studierende stimmten zu, die Interaktion mit den Dozierenden vermisst zu haben. Des Weiteren vermissten 80 (56,7 %) Studierende die Interaktion mit den Kommilitoninnen und Kommilitonen, und 21 (14,9 %) stimmten zu, lieber in einer Gruppenvorlesung zu lernen als alleine. Insgesamt gaben 27 (19,1 %) Studierende an, dass sie sich bei der Bearbeitung von DAVL leicht ablenken lassen, 39 (27,7 %) konnten sich höchstens teilweise konzentrieren.

Die Studie zeigt, dass 132 (94,3 %) Studierendedas Format der DAVL für die Vermittlung von Theorie für geeignet halten. Demgegenüber empfinden 99 (70,7 %) Studierende DAVL als nicht geeignet, um praktische Fertigkeiten zu vermitteln. Insgesamt antworteten 110 (78,0 %) Studierende, dass digitale Formate wie DAVL ihr selbstständiges Lernen fördern.

**Figure 3:**
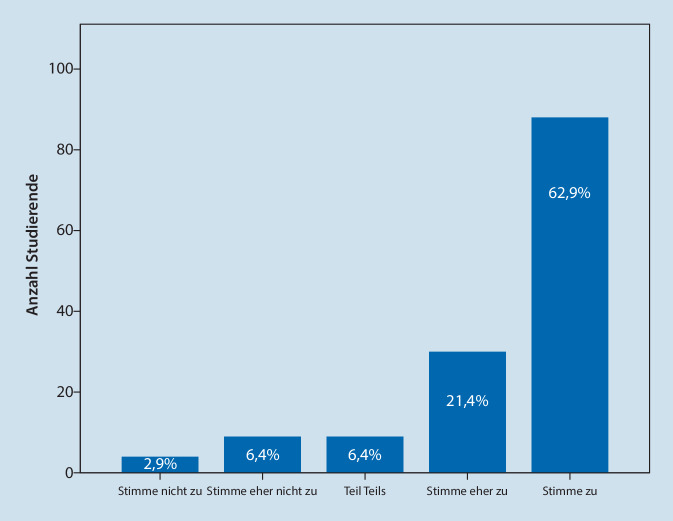

Im Hinblick auf die Zukunft des Lehrformats „Vorlesung“ aus Sicht der Studierenden ergab die Studie, dass 94 (67,2 %) die anästhesiologischen Vorlesungen zukünftig v. a. in digitaler asynchroner Form bevorzugen. Bei einem parallelen Angebot von synchroner Präsenzvorlesung und inhaltsgleicher asynchroner Vorlesung würden 42 (30,0 %) zumindest teilweise eine Präsenzvorlesung besuchen. Abschließend erklärten 118 (84,3 %) Studierende, auch zukünftig mehr DAVL zu bearbeiten, als es bei Präsenzvorlesungen der Fall wäre (Abb. 3).

### Video-Online-Seminare

Im Rahmen der Umfrage wurden die freiwilligen VOS von 63 (67,7 %) der 93 teilnehmenden Studierenden der Semester 7–9 besucht. Technische Probleme traten kaum auf. Ein Anteil von 55 (87,3 %) Studierenden war mit der angebotenen Interaktion zufrieden. Die Interaktion, beispielsweise über den Chat, führte dazu, dass sich 26 (41,2 %) stärker aktiv beteiligten als in einem Präsenzseminar. Positiv wurde bewertet, dass die Dozierenden durch das Einbinden von Quizfragen und Abstimmungen das Wissen der Studierenden gut einschätzen und auf Lücken eingehen konnten bzw. bekannte Inhalte verkürzt behandelten. VOS werden von 54 (85,7 %) Studierenden als sinnvolle Plattform zur Wissensvermittlung und Fallbesprechung eingeschätzt. Praktische Fertigkeiten können allerdings auch über VOS nur begrenzt vermittelt werden. Insgesamt wünschen sich 39 (62,9 %) Studierende zukünftig weitere VOS.

### Praxisunterricht

Das eLearning-Programm wurde von 48 (92,3 %) der 52 an der Studie teilnehmenden Studierenden der Semester 7 und 8 genutzt, um sich mit Lehrvideos und Theorieinhalten auf Praktika vorzubereiten. Das Programm wurde von den Studierenden als sehr hilfreich für die Vermittlung von Theorie sowie die Demonstration von Patientenkommunikation beschrieben, so dass 16 (20,8 %) Studierende angaben, durch das eLearning Gesprächstechniken für die Patientenkommunikation erlernt zu haben. Nichtsdestotrotz antworteten 34 (70,8 %) Studierende, dass ein eLearning-Programm den Patientenkontakt nicht ersetzen kann.

Die Studierenden haben in den freien Kommentaren die Erfahrungen im Praktikum besonders hervorgehoben. Für die Zukunft wünschen sich 132 (94,2 %) mindestens teilweise mehr Präsenzveranstaltungen für die Praxis in Kleingruppen. Insgesamt gaben 92 von 112 (82,1 %) Studierenden an, dass sie sich mit der digitalen Lehre besser auf die praktischen Präsenzveranstaltungen vorbereiten, als es bei Präsenzvorlesungen der Fall wäre. Bei stattgefundenen Praktika konnten 72 von 106 Studierenden (67,9 %) durch die digitale Vorbereitung einen höheren subjektiven Lerngewinn erzielen.

## Diskussion

Die Ergebnisse der Studie vermitteln einen Einblick in die Chancen und Herausforderungen unterschiedlicher digitaler Lehrformate. Ein besonderes Augenmerk wurde auf das Format der DAVL gelegt, deren Vor- und Nachteile im Folgenden diskutiert werden sollen. Die DAVL entstanden an der Universitätsmedizin Mainz im Fachbereich Anästhesiologie durch eine Neustrukturierung der vorherigen Präsenzvorlesung auf einen digitalen 20-minütigen Vortrag mit der Fokussierung auf wesentliche Lerninhalte. Ein großer Vorteil dieser DAVL ist die enorme Reichweite: 86,5 % der befragten Studierenden haben mindestens die Hälfte der DAVL gesehen. Zudem ermöglicht eine kurze Vorlesung, beispielsweise als Impulsvortrag, den Studierenden die Konzentration auf wesentliche Inhalte. Bei der digitalen Bereitstellung ist es dennoch von Bedeutung, ausreichend Material zum Selbststudium zur Verfügung zu stellen [1]. Hierbei wurde von den Studierenden neben den DAVL mit großer Mehrheit das im LMS bereitgestellte begleitende Angebot aus Folien, Quizfragen und vertiefenden Materialien geschätzt. Diese klar strukturierte Lernmanagementsystem-Plattform ist wichtig für eine übersichtliche Präsentation der Materialien [5]. Dabei sollten neben einer Strukturierung Schwerpunkte gesetzt und die Reihenfolge und Wichtigkeit der Themen für die Studierenden verdeutlicht werden [16]. Diese Qualitätskriterien wurde in der vorliegenden Studie bei der Erstellung der digitalen Kursinhalte berücksichtigt. In unserer Studie schätzten 95,0 % der teilnehmenden Studierenden, eine strukturierte Übersicht der Kursthemen im LMS. Eine weitere Option zur Übersicht ist die Dokumentation des persönlichen Lernfortschritts durch die Studierenden [21]. Zudem besteht digital die Möglichkeit, Material schnell zu aktualisieren [10]. Darüber hinaus bietet digitales Lehrmaterial den Studierenden die Möglichkeit, Lehrinhalte aus vorherigen Semestern zur Wiederholung und zur Klausurvorbereitung erneut nutzen zu können. Hiervon machten 72,7 % der Studierenden der Universitätsmedizin Mainz, die den Fragebogen ausfüllten, Gebrauch. Dies deckt sich mit den Ergebnissen einer Studie, in der 70 % von 223 analysierten Studierenden das online verfügbare Unterrichtsmaterial vergangener Semester wiederholt konsultierten [14]. Auch können PJ-Studierende in der Anästhesiologie auf die digitalen Lehrmaterialien zurückgreifen und zusätzlich profitieren. Die DAVL ist unter der Voraussetzung, dass die gerade diskutierten Qualitätskriterien erfüllt werden, der Präsenzlehre somit in Struktur, Verfügbarkeit, Beliebtheit und Vermittlung von theoretischem Wissen aus Sicht der Studierenden wahrscheinlich überlegen.

Neben den genannten Vorteilen der Strukturierung und Fokussierung sowie der vielfältigen Einsatzmöglichkeit erlaubt die DAVL gleichzeitig die individuelle Erweiterung der Lerninhalte. So pausierten 67,4 % der antwortenden Studierenden die DAVL, um sich zwischendurch weitere Informationen anzueignen. Eine Untersuchung bei Studierenden der Harvard-Medical-School vor der COVID-19-Pandemie hat gezeigt, dass 88,5 % der dort befragten Studierenden bei zur Verfügung gestellten DAVL videobeschleunigende Technologien benutzten. Im Durchschnitt wurden die Vorlesungen 1,67-fach schneller angesehen als die Originalaufnahme. Die Studierenden gaben an, die Vorlesung so individueller zu bearbeiten und Zeit zu sparen, um beispielsweise zusätzliche Informationen nachzulesen [6]. Diese Vorteile des orts- und zeitunabhängigen Lernens im eigenen Tempo beschrieben auch 95,7 % der Studierenden unserer Studie. So konnten 71,6 % unserer Studienteilnehmenden mittels DAVL die Vorlesungsinhalte umfassender und zum großen Teil schneller verstehen. Die Flexibilität und Zeitersparnis der DAVL werden von weiteren Autoren bestätigt [8, 17]. Zudem erklärten 78,0 % der befragten Studierenden, dass die digitale Lehre ihre Selbstständigkeit gefördert hat [20]. Dies könnte einen positiven Effekt auf das selbstständige lebenslange Lernen und die Eigenverantwortung als Arzt bzw. Ärztin haben. Die genannten Vorteile kommen insbesondere durch die Integration der digitalen Inhalte im Rahmen einer Blended-Learning-Strategie zum Tragen [7]. Somit kann die grundlegende Theorie im Gegensatz zur Präsenzvorlesung mittels DAVL von jedem Studierenden individuell in seinem eigenen Tempo erarbeitet und vertieft werden.

Die beschriebenen positiven Erfahrungen der Studierenden mit der Individualisierung der Vorlesung durch die ständige Verfügbarkeit wirft die Frage nach der Zukunft der Präsenzvorlesung in der medizinischen Lehre auf. In unser Studie halten 94,3 % der untersuchten Studierenden DAVL zur Vermittlung von Theorie für geeignet. Ein weiterer Einflussfaktor ist, dass deutlich mehr Studierende (80,9 %) angaben, die DAVL bearbeitet zu haben, im Vergleich zum Besuch einer Präsenzvorlesung. Hierbei ist zu beachten, dass die in unserer Studie untersuchten DAVL als zeitlich- und inhaltlich überarbeitete Umwandlungen der ursprünglichen Präsenzvorlesungen entstanden. Dies sollte bei der Gegenüberstellung von DAVL mit der analogen Präsenzvorlesung beachtet werden und hat möglicherweise Auswirkungen auf die Antworten der Studierenden. Als Nachteil digitaler asynchroner Formate soll in der Diskussion um die Zukunft der medizinischen Vorlesung das Argument bedacht werden, dass Studierende digital im Vergleich zu einer Präsenzvorlesung nur bedingt inspiriert und für beispielsweise die Anästhesiologie begeistert werden können [2]. Trotz dieses potenziellen Nachteils hätten in unserer Studie 84,3 % der antwortenden Studierenden in Zukunft eine höhere Motivation, die Theorie mittels DAVL zu erlernen als durch Präsenzvorlesungen. Dies entspricht den Ergebnissen einer Studie aus Israel, die gezeigt hat, dass Studierende die Rückkehr zur reinen Präsenzvorlesung nach der Pandemie nicht befürworten [23]. Hier liegt ein großes Potenzial, die überwiegende Mehrheit der Studierenden im Rahmen der DAVL zu erreichen und für die Anästhesiologie zu begeistern. Jeder Studierende erhält so unabhängig von Vorlesungszeiten oder privaten Verpflichtungen nicht nur die Möglichkeit, von Dozierenden in die Theorie eingeführt zu werden, sondern auch Anreize zur weiteren Beschäftigung mit den ausgewählten Themen. Cardall et al. formulieren als Denkanstoß: „Was wäre verloren, wenn die Präsenzvorlesung der Dinosaurier der medizinischen Ausbildung wird?“ [6].

Als größte Herausforderungen im Zusammenhang mit DAVL ist neben der fehlenden Interaktion mit den Lehrenden und Kommilitoninnen und Kommilitonen auch das reduzierte gegenseitige Feedback zu nennen [17]. In unser Studie gaben 35,5 % der Studierenden an, dass sie zumindest teilweise gerne Rückfragen zu asynchronen Materialien gestellt hätten. Die eingeschränkte Interaktion von Lehrenden und Studierenden kann sich auch auf das studentische Erleben von ärztlichen Rollenvorbildern in der Vorlesung auswirken. Umso wichtiger sind die Bedeutung und Anwendung von Interaktion und Feedback in Seminaren, die entweder online oder präsent stattfinden, sowie das Vorhandensein von klinischen Praktika soweit möglich [20]. Inwieweit bei VOS eine Interaktion entsteht, scheint sehr von der Gestaltung des Seminars abzuhängen. Während manche Autoren interaktive Diskussionen im Rahmen der digitalen Lehre beschreiben [4] und die Interaktionsqualität wie in unserer Studie mit 87,3 % als hoch beschrieben wird, ist die Beteiligung der Studierenden unterschiedlich [23]. 41,2 % unserer Studierenden gaben an, sich bei den VOS z. B. durch das Einbeziehen von Abstimmungen, Quizfragen oder den Chat stärker zu beteiligen als bei einer vergleichbaren Präsenzveranstaltung. Eine zusätzliche Möglichkeit ist das Vorbereiten von Patientenfällen, u. a. anhand von Videos, die anschließend in VOS besprochen werden [22]. Die Kombination von DAVL für die Vermittlung theoretischer Inhalte und die VOS zur Wissensvertiefung scheint sich hier sehr gut zu ergänzen.

Zentrale Punkte der medizinischen Ausbildung sind der Patientenkontakt und die Kommunikation mit dem Patienten. Das Lernen am und vom Patienten kann in der digitalen Lehre nur bedingt abgebildet werden [4, 8]. So gaben 20,8 % unserer Studierenden an, ihre Patientenkommunikation im Rahmen der eLearning-Programme verbessert zu haben. Folglich können eLearning-Programme das Erlernen der Patientenkommunikation unterstützen, aber wie 70,8 % der Studienteilnehmenden angeben, nicht ersetzen. In einer Situation wie der COVID-19-Pandemie, in der der Einbezug von Studierenden in praktische klinische Tätigkeiten gering bis kaum möglich ist, gibt es Ansätze, den verringerten Patientenkontakt durch Optionen der digitalen Lehre abzumildern. Dazu gehört beispielsweise der Einsatz von VOS, um in kleinen Gruppen Fallbeispiele zu besprechen sowie Anamnesetechniken an virtuellen und realen Patienten zu üben [15, 19]. Insbesondere in Bezug auf die zukünftig verstärkte digitale Prägung des ärztlichen Berufslebens, beispielsweise durch den Einsatz von Videosprechstunden, ergeben sich neue Möglichkeiten der digitalen Patienteninteraktion, die in die medizinische Lehre integriert werden können.

Die Diskussion der verschiedenen digitalen Formate stellt die aktuellen Chancen und Herausforderungen der medizinischen Lehre heraus und wirft die Frage nach der zukünftigen Gestaltung der Lehre auf. Vor allem in einer Postpandemiephase bietet die Strategie von Blended-Learning-Lehrformaten eine große Chance [7, 10, 11]. Vorlesungen könnten beispielsweise zum Großteil in Form der nun etablierten DAVL angeboten werden und den Studierenden die Möglichkeit bieten, sich im eigenen Tempo zeit- und ortsunabhängig auf Präsenzveranstaltungen vorzubereiten. Zusätzlich können VOS und eLearning-Module den vorbereitenden Wissenserwerb sinnvoll ergänzen. In den darauffolgenden Präsenzveranstaltungen kann der Fokus v. a. auf die Vertiefung, Diskussion und Anwendung des bereits angeeigneten Wissens gesetzt werden [12]. Dieses Konzept wird als „inverted classroom“ bezeichnet. Die Verlagerung der Vorlesung an einen individuell gewählten Zeitpunkt kann neue Zeitfenster im Stundenplan für die Vertiefung und Verfestigung der Theorie als Vorbereitung für den (praktischen) Kleingruppenunterricht schaffen.

Die Beschäftigung mit den Auswirkungen der aktuellen digitalen Lehre ermöglicht es, die Zukunft der medizinischen Lehre unter Berücksichtigung der verschiedenen Aspekte zu adaptieren und zu optimieren. Hierzu haben wir mit der vorliegenden Umfrage einen relevanten Beitrag leisten können. Als eine Limitation unserer Studie ist zu nennen, dass nur Studierende der Universitätsmedizin Mainz befragt wurden. Eine vergleichende deutschlandweite Studie würde die Forschungsansätze vertiefen. Insgesamt haben 18,4 % der angeschriebenen Studierenden an der Studie teilgenommen. Diese Rücklaufquote ermöglicht keine uneingeschränkte Verallgemeinerung der Ergebnisse auf alle Studierenden und ist daher in der Analyse kritisch zu berücksichtigen. Zur Ergänzung der einmalig erhobenen Antworten könnten Folgebefragungen stattfinden, um mögliche Veränderungen im Pandemieverlauf und eine Gewöhnung an digitale Formate zu erfassen. Interne Qualitätsumfragen und ein ständiges Monitoring der Lehrentwicklung haben zukünftig eine besondere Bedeutung [2]. Die große Herausforderung ist die Exploration, zu welchen Anteilen digitale Lehre und Präsenzlehre für einen bestmöglichen Lernerfolg eingesetzt werden können [13, 21]. Es bleibt abzuwarten, welche Potenziale die zunächst „notfallbedingte Fernlehre“ [18] langfristig freisetzen wird.

## Fazit für die Praxis

- Digital aufgezeichnete Vorlesungen (DAVL) bieten ein großes Potenzial für die zukünftige medizinische Lehre, da eine größere Anzahl an Studierenden im Vergleich zu einer Präsenzvorlesung erreicht werden kann.
- Asynchrone Lehrmaterialien wie DAVL geben den Studierenden die Möglichkeit, zeit- und ortsunabhängig selbstbestimmt zu lernen.
- Digitale Video-Online-Seminare können Studierende interaktiv einbinden und in Zeiten sozialer Distanzierung einen wichtigen Beitrag zur Wissensvermittlung und der Bearbeitung von Fallbeispielen und Lehrvideos leisten.
- Präsenzpraktika werden von den Studierenden als sehr wichtig empfunden und sollten, wenn möglich, in Kleingruppen stattfinden.
- Langfristig bietet das Konzept des Blended Learning die Möglichkeit, individuell als geeignet empfundene Formate der Digital- und Präsenzlehre zu kombinieren.
- Die COVID-19-Pandemie bietet als didaktische Herausforderung die Chance für eine Weiterentwicklung der medizinischen Lehre.

## Abkürzungen

COVID-19: Coronavirus disease 2019
DAVL: Digital aufgezeichnete Vorlesung
LMS: Lernmanagementsystem
SARS-CoV-2: Severe acute respiratory syndrome coronavirus type 2
VOS: Video-Online-Seminar
WHO: World Health Organization
